# Assignment of IVL-Methyl side chain of the ligand-free monomeric human MALT1 paracaspase-IgL_3_ domain in solution

**DOI:** 10.1007/s12104-022-10105-3

**Published:** 2022-09-12

**Authors:** Xiao Han, Maria Levkovets, Dmitry Lesovoy, Renhua Sun, Johan Wallerstein, Tatyana Sandalova, Tatiana Agback, Adnane Achour, Peter Agback, Vladislav Yu. Orekhov

**Affiliations:** 1 Science for Life Laboratory, Department of Medicine, Karolinska Institute, and, Division of Infectious Diseases, Karolinska University Hospital, 171 76 Stockholm, Sweden; 2 Department of Chemistry and Molecular Biology, University of Gothenburg, Box 465, 40530 Gothenburg, Sweden; 3 Department of Structural Biology, Shemyakin-Ovchinnikov, Institute of Bioorganic Chemistry RAS, Moscow, Russia 117997; 4 Department of Molecular Sciences, Swedish University of Agricultural Sciences, Box 7015, 750 07 Uppsala, Sweden; 5 Swedish NMR Centre, University of Gothenburg, Box 465, 40530 Gothenburg, Sweden

**Keywords:** MALT1, Paracaspase, ^1^H, ^13^C Ile, Val, Leu-Methyl resonance

## Abstract

Mucosa-associated lymphoid tissue protein 1 (MALT1) plays a key role in adaptive immune responses by modulating specific intracellular signalling pathways that control the development and proliferation of both T and B cells. Dysfunction of these pathways is coupled to the progress of highly aggressive lymphoma as well as to potential development of an array of different immune disorders. In contrast to other signalling mediators, MALT1 is not only activated through the formation of the CBM complex together with the proteins CARMA1 and Bcl10, but also by acting as a protease that cleaves multiple substrates to promote lymphocyte proliferation and survival via the NF-κB signalling pathway. Herein, we present the partial ^1^H, ^13^C Ile/Val/Leu-Methyl resonance assignment of the monomeric apo form of the paracaspase-IgL_3_ domain of human MALT1. Our results provide a solid ground for future elucidation of both the three-dimensional structure and the dynamics of MALT1, key for adequate development of inhibitors, and a thorough molecular understanding of its function(s).

## Introduction

**Fig. 1:**
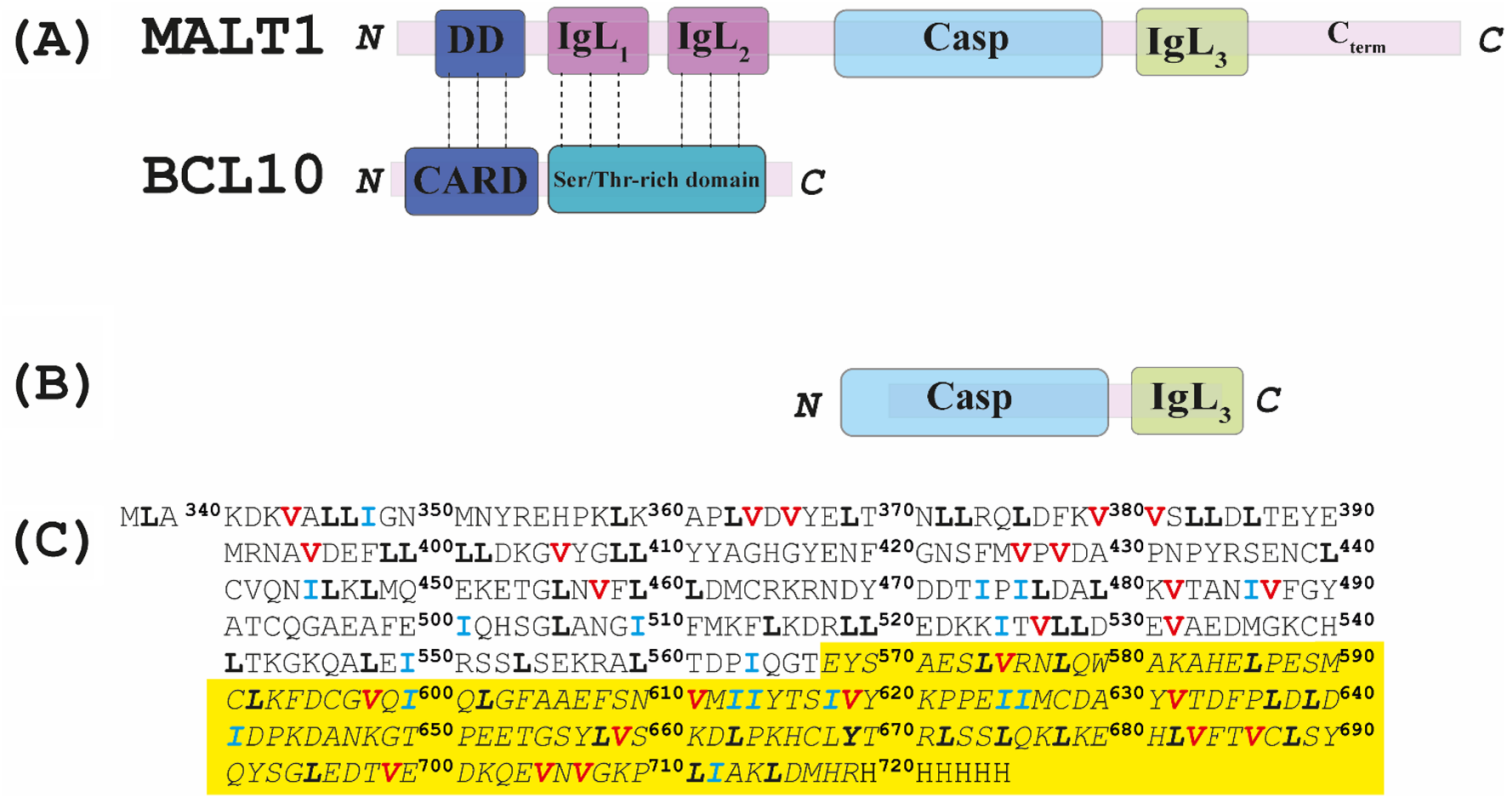
Domain organization. **A** Schematic representation of the oligomer complex formed by MALT1 and BCL10. MALT1 comprises five domains including the N-terminal DEATH domain (DD), two immunoglobulin-like domains (IgL_1_ and IgL_2_), the caspase-like domain (Casp) and a third immunoglobulin-like domain (IgL_3_) **B** Schematic representation of the MALT1(Casp-IgL_3_)_338–719_ self-folding unit that was used within the present study. **C** Sequence and numbering of human MALT1(Casp-IgL_3_)_338–719_ domains in which the IgL_3_ domain is highlighted and typed in italic. The C-terminal his-tag is also depicted. The amino acids Ile, Leu and Val are labelled in blue, bold black and red, respectively

MALT1 has been identified as a key player in intracellular pathways that lead to the activation of the transcription factor NF-κB which ultimately controls the development and proliferation of T and B cells (Ruland et al. 2003; Ruefli-Brasse et al. 2003; Jaworski et al. 2014; Gewies et al. 2014; Bornancin et al. 2015; Juilland and Thome 2018; Schlauderer et al. 2018; Gehring et al. 2018; Hailfinger et al. 2009; Dunleavy and Wilson 2014; Lenz, 2015; Uren et al. 2000). The function of MALT1 is triggered upon activation of B- or T-cell receptors, as well as NK cells through interactions with Fc receptors (Rosebeck et al. 2011). Dysfunctions in these MALT1-directed pathways are coupled to the potential development of aggressive lymphomas with high resistance to current chemotherapies, as well as to the initiation of an array of immune disorders (Solsona et al. 2022) Full length MALT1 is composed of five domains (Hailfinger et al. 2009) including the N-terminal death domain (DD), two immunoglobulin-like domains (IgL_1_ and IgL_2_), the paracaspase or caspase-like domain (Casp) and a third immunoglobulin-like domain (IgL_3_), followed by an unstructured C-terminal tail domain (Fig. 1A). The triggering of activating receptors from both innate and adaptive immune responses induces the formation of CARMA-BCL10-MALT1 (CBM) complexes (Ruland and Hartjes 2019). Indeed, CBM formation is pivotal for the adequate activation of the NF-κB transcription factor. The DD domain of MALT1 binds to the core of the BCL10 filament through interactions with the caspase activation and recruitment domain (CARD) of BCL10 (Schlauderer et al. 2018), while additional interactions are also formed between the IgL_1_ and IgL_2_ domains of MALT1 and the Ser/Thr rich domain of BCL10 (Langel et al. 2008) (Fig. 1A). It should be noted that the C-terminal section of MALT1, which comprises the paracaspase and the IgL_3_ domains, is most probably protruding out from the BCL10 filament, although its structure could not be detected due to high flexibility (Schlauderer et al. 2018) Thus, the molecular and dynamic bases underlying the potential allosteric modulation of the function of this section of MALT1 remain in our opinion unknown.

Importantly, it has been demonstrated that the regulating function of MALT1 on NF-κB can be exerted by at least two routes, one of which includes the protease activity acquired by MALT1 upon participating in the formation of the CBM complex (Che et al. 2004; Solsona et al. 2022; Rebeaud et al. 2008; Coornaert et al. 2008). However, it should be noted that MALT1 promotes a second route for NF-κB activation by acting as a scaffold when bound to BCL10, recruiting E3 ubiquitin ligases, such as TRAF6 and the linear ubiquitin chain assembly complex (LUBAC), which ultimately results in ubiquitination of BCL10 and MALT1 (Sun et al. 2004; Yang et al. 2014; Deng et al. 2000; Oeckinghaus et al. 2007). It has been previously demonstrated that activation of MALT1 requires the monoubiquitination of residue K644 on the surface of the IgL_3_ domain (Fig. 1A) (Pelzer et al. 2013). More recent data suggested that ubiquitination of the IgL_3_ domain may induce conformational changes that could be allosterically communicated to the active site of the paracaspase domain of MALT1 (Schairer et al. 2020).

Crystal structures of individual MALT1 domains and combinations thereof in complex with allosteric ligands have been previously determined (Yu et al. 2011; Eitelhuber et al. 2015; Schlauderer et al. 2013). Furthermore, the recently developed AlfaFold prediction server provides an excellent source of reliably predicted three-dimensional structures of proteins and protein domains (Jumper et al. 2021), including human full-length MALT1 in monomeric form. However, although crystal structures provide crucial atomic-scale information about the three-dimensional fold of proteins as well as exquisite architectural details of e.g. catalytic sites, they still represent snapshots of energy minimized states and can thus seldom provide adequate information for e.g. establishing the dynamic bases underlying allosteric communication. Noteworthy, to the best of our knowledge, the three-dimensional structure of the apo monomeric form of the human MALT1(Casp-IgL_3_)_338–719_ in solution has remained missing and all available crystal structures of MALT1 are dimer (Yu et al. 2011; Wiesmann et al. 2012). In contrast, NMR spectroscopy can provide much more ample information about both domain and local conformational flexibilities. It has been previously demonstrated that the truncated version of MALT1 which comprises only the caspase-like and the IgL_3_ domains MALT1(Casp-IgL_3_)_338–719_ (Fig. 1B, C) retains an active fold (Wiesmann et al. 2012) and that it forms dimers that are functionally important (Hachmann et al. 2012; Wiesmann et al. 2012). Hence, we here focused our efforts on this part of MALT1. We have previously reported the almost complete ^15^ N/^13^C/^1^H backbone assignment of the apo form of the human MALT1 paracaspase region together with the third immunoglobulin-like (IgL_3_) domain by high resolution NMR (Unnerstale et al. 2016). Here, we partially assigned the IVL-Methyl side chains of the ligand-free monomeric human MALT1 paracaspase-IgL_3_ domain in solution.

## Methods and experiments

### Expression and purification of labelled MALT1(Casp-IgL_3_)_338–719_

The DNA sequence encoding for the caspase and IgL_3_ domains of human MALT1, corresponding to residues 338–719 (Fig. 1C) and a C-terminal His6-tag was cloned into pET21b (Novagen). The MALT1_338–719_-his construct was transformed into *Escherichia coli* strain T7 express competent cells and thereafter expressed in different isotopic labelling combinations in ^1^/^2^H, ^15^ N, ^12^/^13^C-labelled M9 medium. Chemicals for isotope labelling (ammonium chloride, ^15^ N (99%), D-glucose, ^13^C (99%), deuterium oxide) were purchased from Cambridge Isotope Laboratories, Inc. Cells were cultivated at 37 ℃ and were induced at an OD_600_ of approximately 0.8 for 16 h at 16 ℃ by addition of β-D-1-thiogalactopyranoside (IPTG) to 0.5 mM final concentration.

For the incorporation of methyl groups with the desired isotopic labelling pattern, alpha-keto acids were added as supplements to M9 medium and they served as biosynthetic precursors. MALT1(Casp-IgL_3_)_338–719_ was expressed in 1 L of D_2_O M9 medium using 3 g/L of U-[^13^C,^2^H]-glucose (CIL, Andover, MA) as the main carbon source and 1 g/L of ^15^NH_4_Cl (CIL, Andover, MA) as the nitrogen source. One hour prior to induction, precursors were added to the growth medium as previously described (Tugarinov et al. 2006). For precursors, 70 mg/L alpha-ketobutyric acid, sodium salt (^13^C4, 98%, 3,3-^2^H, 98%) and 120 mg/L alpha-ketoisovaleric acid, sodium salt (1,2,3,4-^13^C4,99%, 3, 4, 4, 4, -^2^H 97%) (CIL, Andover, MA) were used. Bacterial growth was continued for 16 h at 16 °C and the cells were thereafter harvested by centrifugation.

Cells were resuspended in lysis buffer 20 mM TrisHCl (pH7.6), 150 mM NaCl, 2 mM DTT and lysed using ultra-sonicator, followed by centrifugation at 40,000 g for 30 min to remove cell debris. The supernatant was collected and incubated with Ni^2+^ Sepharose 6 Fast Flow (GE Healthcare) for 1 h at 4 ℃. The target protein was eluted with lysis buffer containing 200–500 mM imidazole. A Q-Sepharose HP column (GE Healthcare) was used to separate the monomeric MALT1(Casp-IgL_3_)_338–719_ protein from the dimer form. A final size exclusion chromatography (SEC) step using a HiLoad 16/600 Superdex 200 prep grade column (GE Healthcare) was performed, with running buffer 20 mM HEPES 7.4, 50 mM NaCl, 1 mM DTT. The final monomer MALT1(Casp-IgL_3_)_338–719_ protein sample was subsequently exchanged to a buffer (10 mM Tris 7.6, 50 mM NaCl, 2 mM TCEP, 0.002% NaN3) suitable for NMR experiments using gravity flow PD10 desalting columns (GE Healthcare). Final yields from a four litres M9 culture were approximately 8 mg of purified protein. Purified monomeric MALT1(Casp-IgL_3_)_338–719_-his was concentrated to at least 0.4 mM for NMR data acquisition.

## NMR spectroscopy

NMR spectra were recorded at 298 K and 308 K on 700 MHz (Bruker AVANCE III) or on 800 MHz, 900 MHz (Bruker AVANCE III-HD) spectrometers equipped with cryo-enhanced 5 mm QXI, 3 mm TCI, and 3 mm TCI probes, respectively. 2D ^1^H-^15^ N Best-TROSY-transverse relaxation optimized spectroscopy (TROSY) was used (Eletsky et al. 2001; Pervushin et al. 1997; Schulte-Herbruggen and Sorensen 2000). Three dimension (3D) Best-TROSY type HNCO and 3D HNCA experiments were collected using iterative non-uniformly sampling (NUS) (Favier and Brutscher 2011). Deuterium decoupling was applied in 3D Best-TROSY HNCA. The assignment of the ^1^H, ^13^C Methyl Val, Leu, Ile amino acids of MALT1(Casp-IgL_3_)_338–719_ was based on a set of 3D resonance experiments including HMCM(CGCB)CA and HMCM(CGCBCA)CO for Ile/Leu and HMCM(CB)CA for Val residues. The pulse programs were identical to hmcmcbcagpwg3d and hmcmcbcacogpwg3d in Bruker TopSpin3.6 except that methyl HMQC instead of HSQC and ^2^H decoupling were applied (Tugarinov et al. 2014) and 1.8 ms IBurp1 pulse was used for selective inversion of CG2 of Ile.

Intramolecular amide- methyl, NH-CH_3_, interactions were verified through observing cross peaks in 3D SOFAST (SF), ^1^H–^15^ N TROSY NOESY experiments. Additional intramolecular Methyl-Methyl interactions were obtained from 4D ^13^C,^13^C-SF HMQC NOESY (Zwahlen et al. 1998) and 3D ^1^H^13^C^13^C^1^H-TOCSY(Kay et al. 1993) experiments.

The experimental parameters for acquisition in the 2D/3D/4D experiments are summarised in Table 1.

**Table 1:** List of acquisition parameters used for NMR experiments

Experiments	Maximum evolution time, (ms)/ carrier frequency (ppm)/sweep width (ppm)			D1s	Scans	NUS points	NUS %	Time (h)
	F3	F2	F1					
^1^H-^15^ N Best-TROSY^a,c^	9.4(^1^H)/ 4.7/12	38.9(^15^ N)/ 118.0/36.0	–	0.8	4	–	–	1.0
3D Best-TROSY-HNCO^a,f^	79.9(^1^H)/ 4.7/16.0	34.3(^15^ N)/ 118.0/36.0	19.9(^13^C)/ 173.0/15.0	0.5	16	720	12	6.2
3D Best-TROSY –HNCA_2H^a,b^	106.5(^1^H)/ 4.7/12.0	24.0(^15^ N)/ 118.0/36.0	42.4(^13^C)/ 54.0/30.0	0.5	16	2400	13.4	32.4
3D ^1^H–^15^ N SF- NOESY-TROSY^a^	79.9(^1^H)/4.67/16.0	27.4(^15^ N)/118/36.0	28.4(^1^H)/4.67/11.0	0.5	16	4600	23	68
4D ^13^C,^13^C-SF-HMQC NOESY-HMQC^c^	F481.0(^1^H)/4.7/14.0	F3/F29.8(^13^C)/ 17.0/18.0	F119.7(^1^H)/4.7/1.8	0.7	8	5400	10.5	84
^1^H^13^C^13^C^1^H-TOCSY^g^	90.9(^1^H)/4.67/1616.0	4.5(^13^C)/39/80 36.0	22.7(^1^H)/4.67/8 11.0	1.0	4	–	–	40
^1^H-^13^C HMQC^a,c^	94.6(^1^H)/4.7/12.0	22.5(^13^C)17.0/20.0	–	1.0	8	–	–	0.5
HMCM(CGCBCA)CO_2H^a,b,d,f^	91.8(^1^H)/ 4.7/14.0 4.74.7	13.1(^13^C)/16.0/16.0	28.9(^13^C)/ 171.0/11.0	1.0	16	1612	60	37.4
HMCM(CGCB)CA_2H^a,b,d^	91.8(^1^H)/ 4.7/14.0 4.74.7	13.1(^13^C)/16.0/16.0	31.8(^13^C)/ 39/20.0	1.0	16	1182	22	27
HMCM(CB)CA_2H^a,b,e^	91.8(^1^H)/ 4.7/14.0 4.74.7	13.1(^13^C)/16.0/16.0	31.8(^13^C)/39.0/20.0	1.0	16	1720	32	38.4

^a^Experiments performed on an 800 MHz spectrometer

^b^Experiments performed with deuterium decoupling

^c^Experiments on 900 MHz spectrometer

^d^Optimized for Ile and Leu

^e^Optimized for Val

^f^T = 308 K

^g^Experiments performed on an 700 MHz spectrometer

The 3D NUS methyl related experiments were processed using NMRpipe (Delaglio et al. 1995) and the IST algorithm in the mddnmr software (Kazimierczuk and Orekhov 2011; Mayzel et al. 2014). The decoupling of the homonuclear one-bond ^13^C^α^-^13^C^β^ scalar coupling in the HNCA, HMCM(CB)CA, and the HMCM(CGCB)CA experiments was performed by deconvolution (Kazimierczuk et al. 2020). The ^1^H, ^13^C and ^15^ N chemical shifts were referred to DSS-_d6_. The ^13^C and ^15^ N chemical shifts were referenced indirectly. The backbone chemical shifts of MALT1(Casp-IgL_3_)_338–719_, ^1^HN, ^15^ N, ^13^C^α^, ^13^C^β^ and ^13^C´ nuclei, have been previously assigned by us (Unnerstale et al. 2016) using the Target Acquisition approach (Isaksson et al. 2013; Jaravine and Orekhov 2006; Jaravine et al. 2008), and can be found in the Biological Magnetic Resonance Data Bank (Ulrich et al. 2008) (http://www.bmrb.wisc.edu/) with the BMRB accession code 25,674. All analyses were performed manually in CcpNmr Analysis 3.0.4 (Vranken et al. 2005). For visualization of the results of Methyl’s assignment on the MALT1(Casp-IgL_3_)_338–719_ model the UCSF Chimera package (Pettersen et al. 2004) was used. The model was created based on the crystal structure of MALT1 (PDB ID: 3V55) and adding missing loops according to the comparative protein modelling approach(Sali & Blundell 1993).

## Extent of assignments and data deposition

**Fig. 2:**
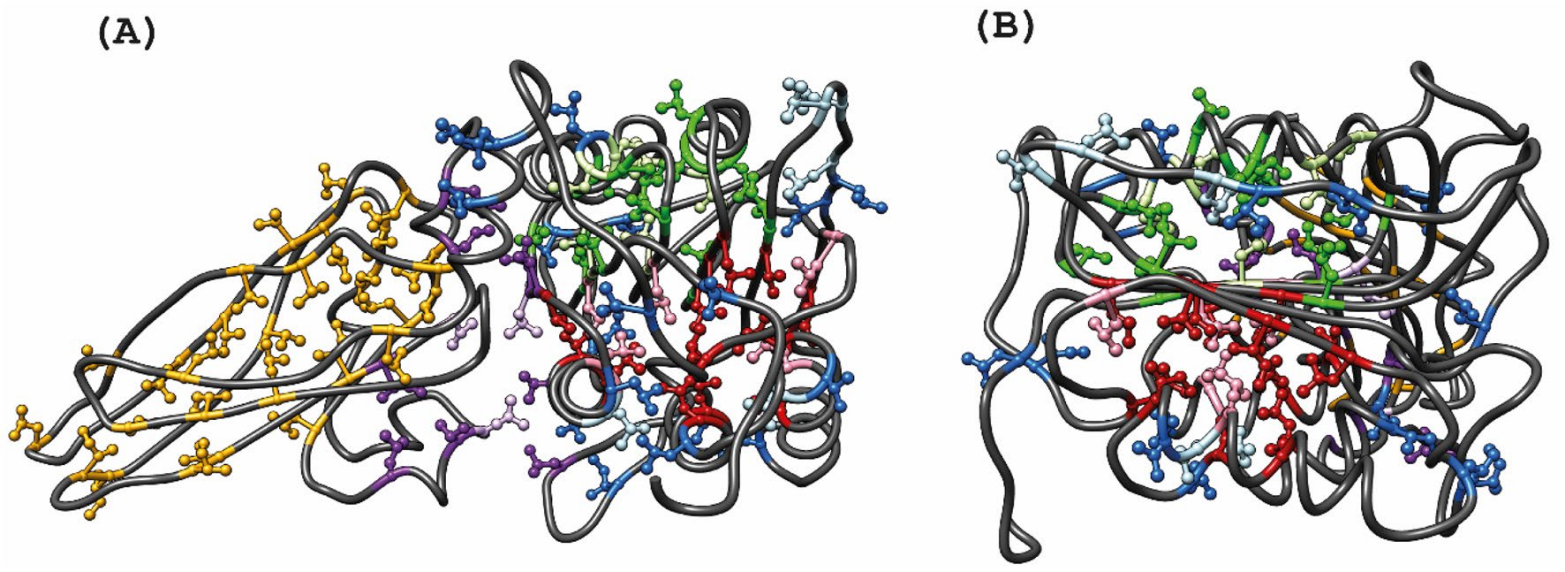
Annotation of the Methyl groups assignment in the MALT1. **A** Four large hydrophobic clusters of methyl Ile, Val, Leu are coloured by: (I) yellow in IgL_3_ domain, (II) violet, between IgL_3_ and paracaspase domains, (III) and (IV) green and red for clusters located on both sides of the beta sheets in the paracaspase domain. **B** 90°-rotated projection of the paracaspase domain only showing (III) and (IV) hydrophobic clusters located around the beta sheets. The methyls of Ile, Val and Leu residues that are lying outside of the hydrophobic cores of MALT1 are coloured in blue. The assigned methyl groups of the amino acids are marked by dark colours corresponding to the clusters and the unassigned residues are coloured in corresponding light colours

Thorough knowledge of both backbone and side chain chemical shift nuclei is important for a complete description of the structural features of the human MALT1(Casp-IgL_3_)_338–719_ complex. We have previously reported the ^15^ N/^13^C/^1^H backbone assignment of the apo form of MALT1(Casp-IgL_3_)_338–719_ in solution (Unnerstale et al. 2016). Methyl-specific isotope labelling has been recently developed as a powerful tool to study the structure, dynamics and interactions of large proteins and protein complexes by solution-state NMR (Tugarinov et al. 2006; Rosenzweig and Kay 2014). Four large hydrophobic clusters assembled by methyl groups of Ile, Leu, Val amino acids could be distinguished in the structure of MALT1(Casp-IgL_3_)_338–719_ (Fig. 2). The first cluster (I) is located mainly in IgL_3_ domain, while the second cluster (II) is localized between the IgL_3_ and Casp (Fig. 2A). The third (III) and fourth (IV) clusters are structural parts of the Casp domain and are located on both side of beta sheets (Fig. 2B).

In this study, we focused on the assignment of the methyl resonances for the side chains of valine (Val), leucine (Leu) and isoleucine (Ile) amino acid residues in the human MALT1(Casp-IgL_3_)_338–719_ construct. The assignment of the ^1^H and ^13^C resonances of methyl group in NMR spectra of large proteins remains a challenge. We therefore used a combination of two highly efficient and complementing protocols. We started with the conventional approach, where the methyl resonances were connected to the known backbone assignments using methyl out-and-back experiments (Tugarinov et al. 2014). Then, the methyl assignments were validated and further expanded using the second approach based on Nuclear Oberhausen Effect (NOE) cross-peak data, peak residue type classification and a known 3D structure or a reliable structural model (Rossi et al. 2016; Pritišanac et al. 2019; Nerli et al. 2021).

## Assignment of ^1^H, ^13^C resonances for methyl Ile, Leu and Val residues in human MALT1(Casp-IgL_3_)_338–719_ through Methyl -C^α^/or C′ correlation

**Fig. 3:**
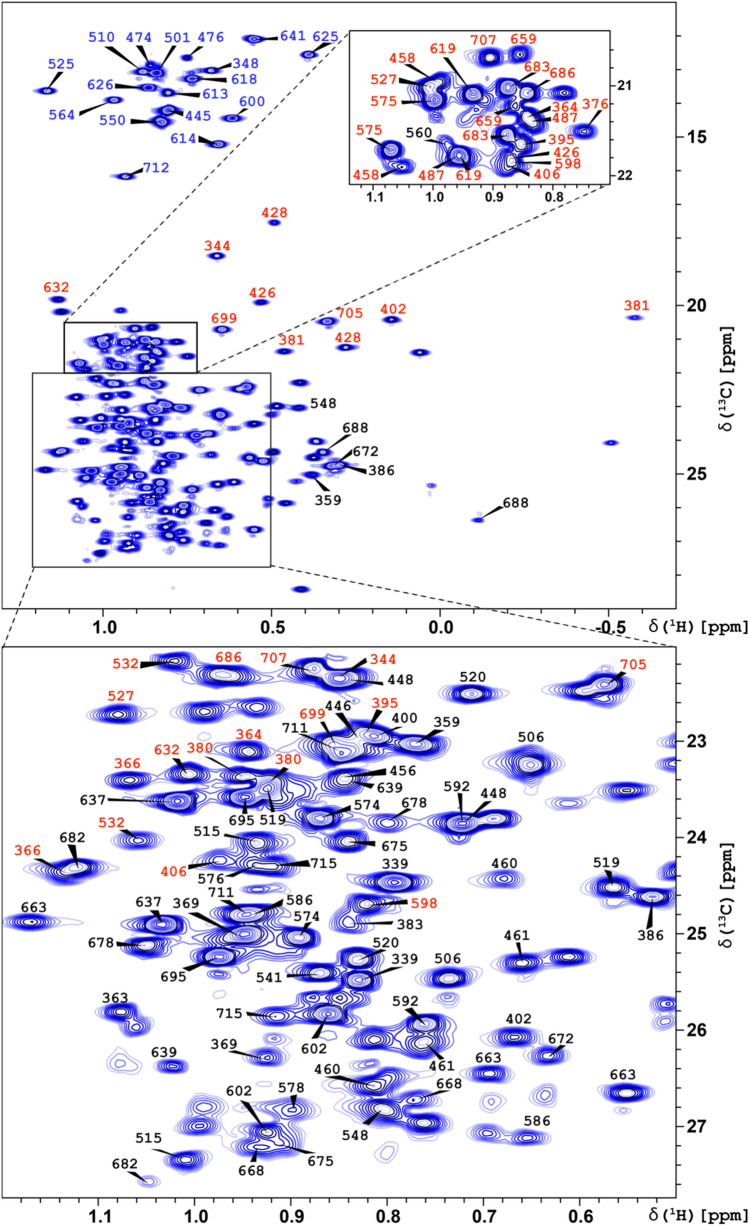
Annotated ^1^H,^13^C-HMQC spectrum of monomeric human apo-MALT1(Casp-IgL_3_**)**_338–719_ Assignments of the cross peaks are depicted by numbers of the corresponding amino acid residues in the protein sequence. Numbers for Ile, Val and Leu are coloured in blue, red and black, respectively. The two insets enlarge the most crowded regions of the spectrum

Our initial approach was based on sets of previously developed experiments (Tugarinov and Kay 2003), where interactions between ^1^H/^13^C labelled methyl groups of Ile, Val and Leu residues, and C^α^ or C′ nuclei in triple, ^2^H, ^13^C, ^15^ N, labelled MALT1(Casp-IgL_3_)_338–719_ protein were monitored. A higher resolution was achieved through NUS acquisition in indirect detection (Table 1). Combination of the previously obtained backbone assignment (Unnerstale et al. 2016) and chemical shifts for C^α^ and C’ from the out-and-back methyl experiments (Table 1) allowed us to assign 10 (out of total 18) Ile, 12/108 Leu and 15 (of 52) Val methyl groups. The assignment at this stage was incomplete because of the relatively low sensitivity of the methyl out-and-back 3D experiments, which lack cross-peaks for a number of methyl signals observed in 2D ^1^H-^13^C HMQC (Fig. 3). The apparent reason for this low sensitivity is fast relaxation of the ^1^H and ^13^C nuclei involved in the magnetization transfer. In addition, the Casp domain is apparently involved in a slow dynamic process leading to line broadening. The out-and-back HMCM(CGCBCA)CO_2H experiment performed at a higher temperature (308 K) showed higher sensitivity. However, we performed most of the experiments at 298 K, because MALT1(Casp-IgL_3_)_338–719_ is unstable at 308 K or higher temperatures. It should be noted that this type of experiment for large proteins usually shows best performance at high temperature, which therefore limits its application to temperature-stable proteins.

## Assignment of ^1^H, ^13^C resonances for methyl Ile, Leu and Val residues in human MALT1(Casp-IgL_3_)_338–719_ based on NOEs contacts

As a next step, we combined backbone amide and side-chain methyl assigned above with NOEs obtained from NH-Methyl NOE in 3D (^1^H-^15^ N) NOESY and Methyl-Methyl NOE interactions in 4D ^13^C-^13^C NOESY spectrum (Nerli et al. 2021) versus the available spatial structure of MALT1. Comparison of the observed NOE cross peaks and their intensities to the corresponding distances in the crystal structure of MALT1(Casp-IgL_3_)_338–719_ permitted additional assignment of the ^1^H, ^13^C methyl resonances. Pairs of geminal ^13^C^δ1^/^13^C^δ2^ and Val ^13^C^γ1^/^13^C^γ2^ resonances were verified through Methyl-Methyl TOCSY interaction (Kay et al. 1993) in ^1^H^13^C^13^C^1^H-TOCSY experiment.

Figure 3 depicts the ^1^H-^13^C HMQC spectrum with the methyl assignment of MALT1(Casp-IgL_3_)_338–719**.**_ Out of a total of 98 ILV (61 in Casp and 37 in IgL_3_) amino acid residues (only 1 methyl for Ile) we assigned 79 (44 for Casp and 35 for IgL_3_): 88% of Val (13 in Casp and 10 in IgL_3_, coloured in red in Fig. 3), 100% of Ile (10 in Casp and 8 in IgL_3_, coloured in blue in Fig. 3) and 70% of Leu (21 in Casp and 17 in IgL_3_, coloured in black in Fig. 3). The majority of the assigned methyls are located in the IgL_3_ domain and belong to the hydrophobic clusters I and II. Assignment of the remaining methyls in clusters (III) and (IV) was hindered by the incomplete backbone assignment, low sensitivity in the out-an-back spectra, as well as due to substantial overlap of several methyl signals of Leu residues. The methyl chemical shifts have been added to the Biological Magnetic Resonance Data Bank deposition 25,674. (Ulrich et al, 2008) (http://www.bmrb.wisc.edu/).

## Conclusion

We present in this study the partial ^1^H /^13^C Ile/Leu/Val methyl resonance assignments for the apo form of human MALT1(Casp-IgL_3_)_338–719_. This assignment will play a crucial role in elucidation of MALT1(Casp-IgL_3_)_338–719_ structure, dynamics, and allosteric pathways as well as for mapping protein–protein and protein–ligand interaction sites.

## Data Availability

The methyl chemical shifts have been added to the Biological Magnetic Resonance Data Bank deposition 25,674. (Ulrich et al., 2008) (http://www.bmrb.wisc.edu/).
